# ATP Restoration by ATP-Deprived Cultured Primary Astrocytes

**DOI:** 10.1007/s11064-024-04276-9

**Published:** 2024-11-16

**Authors:** Gabriele Karger, Johanna Elisabeth Willker, Antonia Regina Harders, Patrick Watermann, Ralf Dringen

**Affiliations:** 1 Centre for Biomolecular Interactions Bremen Faculty 2 (Biology/Chemistry), University of Bremen, P.O. Box 330440, 28334 Bremen, Germany; 2 Centre for Environmental Research and Sustainable Technologies, University of Bremen, P.O. Box 330440, 28334 Bremen, Germany

**Keywords:** Astrocytes, ATP restoration, Glycolysis, Adenosine, Metabolism, Mitochondria

## Abstract

A high cellular concentration of adenosine triphosphate (ATP) is essential to fuel many important functions of brain astrocytes. Although cellular ATP depletion has frequently been reported for astrocytes, little is known on the metabolic pathways that contribute to ATP restoration by ATP-depleted astrocytes. Incubation of cultured primary rat astrocytes in glucose-free buffer for 60 min with the mitochondrial uncoupler BAM15 lowered the cellular ATP content by around 70%, the total amount of adenosine phosphates by around 50% and the adenylate energy charge (AEC) from 0.9 to 0.6. Testing for ATP restoration after removal of the uncoupler revealed that the presence of glucose as exclusive substrate allowed the cells to restore within 6 h around 80% of the initial ATP content, while coapplication of adenosine plus glucose enabled the cells to fully restore their initial ATP content within 60 min. A rapid but incomplete and transient ATP restoration was found for astrocytes that had been exposed to adenosine alone. This restoration was completely prevented by application of the pyruvate uptake inhibitor UK5099, the respiratory chain inhibitor antimycin A or by the continuous presence of BAM15. However, the presence of these compounds strongly accelerated the release of lactate from the cells, suggesting that the ribose moiety of adenosine can serve as substrate to fuel some ATP restoration via mitochondrial metabolism. Finally, the adenosine-accelerated ATP restoration in glucose-fed astrocytes was inhibited by the presence of the adenosine kinase inhibitor ABT-702. These data demonstrate that astrocytes require for a rapid and complete ATP restoration the presence of both glucose as substrate and adenosine as AMP precursor.

## Introduction

Astrocytes are essential partners of neurons in the brain [1–3]. To maintain their many important functions in the brain, astrocytes require substantial amounts of ATP, especially for processes such as the maintenance of the astrocytic membrane potential [4], neurotransmitter uptake and metabolism [5], ATP-driven export processes [6, 7] as well as the synthesis of energy stores such as glycogen [8, 9] and lipid droplets [10]. Thus, the maintenance of a high cellular ATP content and the continuous regeneration of the consumed ATP are highly important to fuel the astrocytic contributions for normal brain function. Accordingly, astrocytes are, at least in culture, able to metabolize a large variety of endogenous and exogenous substrates to maintain a high ATP level [11–14] and use both cytosolic substrate level phosphorylation and mitochondrial oxidative phosphorylation for ATP regeneration [12, 15].

ATP is regenerated from ADP mainly by cytosolic glycolysis and by mitochondrial oxidative phosphorylation in brain cells. The importance of glycolysis for astrocytic ATP regeneration is clearly demonstrated by the observation that astrocytes can even survive an inactivated mitochondrial respiratory chain in culture and in vivo as purely glycolytic cells [16, 17]. Thus, glycolysis appears to be sufficient to regenerate the needed ATP in astrocytes as long as glucose availability is not limited. On the other hand, glucose-deprived astrocytes maintain a high ATP content for many hours by making use of endogenous energy stores as fuels for mitochondrial oxidative phosphorylation [12]. In addition, the presence of various exogenous substrates can prevent the cellular ATP loss observed for astrocytes during extended glucose-deprivation by serving as substrates for mitochondrial ATP regeneration, including monocarboxylates, fatty acids and several amino acids [13]. The contribution of both glycolysis and oxidative phosphorylation for maintaining a high ATP content is also demonstrated by the partial depletion of the cellular ATP level after inhibition of mitochondrial oxidative phosphorylation in glucose-fed astrocytes [12], by the partial ATP regeneration via mitochondrial processes in astrocytes that had been treated with 2-deoxyglucose [14, 18] and by the severe cellular ATP loss reported for the impairment of both metabolic processes [11, 12, 14, 19–22].

Cultured astrocytes contain a high cytosolic ATP concentration of around 7 mM [12, 14], but only low amounts of ADP and AMP [14, 23–27] that account to around 8% (0.56 mM) and 5% (0.35 mM) of the cytosolic ATP concentration, respectively [14]. This leads to an adenylate energy charge (AEC: ([ATP] + 0.5 [ADP]) / ([ATP] + [ADP]+ [AMP])) [28, 29] of around 0.9 in untreated cultured astrocytes [14, 24, 26, 27]. A rapid loss in cellular ATP content was reported for glucose-deprived astrocytes during coincubations with the mitochondrial respiratory chain inhibitor antimycin A [14]. This loss was accompanied by some increase in cellular ADP and AMP levels, while the sum of the three adenosine phosphates (ATP + ADP + AMP) and the AEC were found severely decreased [14].

Although ATP depletion by compromising metabolic pathways has been well studied for cultured astrocytes, hardly any information is available on the ATP restoration in astrocytes that had been partially depleted of ATP. To our knowledge, only few studies reported such data and described a rather limited potential of ATP depleted astrocytes for ATP restoration [30–32]. In order to gain more information on astrocytic ATP restoration, we first depleted cultured astrocytes of ATP by a 60 min preincubation with the mitochondrial uncoupler BAM15 in glucose-free buffer as recently described [18] and studied then ATP restoration after removal of the uncoupler and application of suitable substrates. ATP-deprived astrocytes almost completely restored their initial ATP level within 6 h after application of glucose in a process that was strongly accelerated by the presence of adenosine. Also, the presence of adenosine in the absence of glucose allowed a concentration-dependent rapid ATP restoration during 60 min, but this restoration was partial, significantly lowered by inhibitors of adenosine metabolism and not persistent during longer incubations. In addition, inhibitors of mitochondrial pyruvate uptake or of the respiratory chain did not affect ATP restoration in the presence of glucose but prevented ATP restoration from adenosine as exclusive substrate. These data demonstrate that glycolytic glucose metabolism enables ATP-depleted astrocytes to rapidly restore ATP in the presence of adenosine as AMP precursor, while ATP restoration from extracellular adenosine alone in the absence of glucose requires mitochondrial processes to make also use of the ribose moiety in adenosine as energy substrate.

## Materials and Methods

### Materials

The adenosine deaminase inhibitor 2’-deoxycoformycin (DCF), adenylate kinase, AMP, antimycin A, BAM15, fetal calf serum (FCS), glucose-6-phosphate, 3-(4,5-dimethyl-2-thiazolyl)-2,5-diphenyl-2 H-tetrazolium bromide (MTT), the redox cyclers phenazine ethosulfate (PES) and phenazine methosulfate (PMS) as well as glucose-free Dulbecco’s modified Eagles medium (DMEM) were obtained from Sigma-Aldrich (Steinheim, Germany). DMEM (containing 25 mM glucose) and penicillin G/streptomycin sulfate solution were purchased from Thermo Fisher Scientific (Schwerte, Germany). Acetate, bovine serum albumin, dimethyl sulfoxide (DMSO), NAD^+^, NADH and NADPH were obtained from AppliChem (Darmstadt, Germany). ATP, glutamate-pyruvate transaminase, lactate dehydrogenase (LDH), pyruvate kinase and yeast glucose-6-phosphate dehydrogenase were purchased from Roche Diagnostics (Mannheim, Germany). Nicotinamide was from Fluka (Steinheim, Germany). The adenosine kinase inhibitor ABT-702 and the purine nucleoside phosphorylase inhibitor forodesine hydrochloride were obtained from MedChemExpress (Monmouth Junction, NJ, USA). The Cell Titer Glo^®^ 2.0 ATP Assay Kit was from Promega (Walldorf, Germany) and UK5099 from Merck (Darmstadt, Germany). All other basal chemicals were obtained from Sigma-Aldrich (Steinheim, Germany), Roth (Karlsruhe, Germany) or Merck (Darmstadt, Germany). Sterile cell culture consumables, unsterile 96-well plates and black microtiter plates were purchased from Sarstedt (Nümbrecht, Germany).

### Astrocyte-Rich Primary Cultures

Primary astrocyte-rich cultures were prepared from the brains of newborn Wistar rats as previously described in detail [33]. The rats were purchased from Charles River Laboratories (Sulzfeld, Germany) and treated in accordance to the State of Bremen, German and European animal welfare acts. The harvested cells were counted and 300,000 viable cells were seeded in 1 mL culture medium (90% DMEM containing 25 mM glucose, 44.6 mM sodium bicarbonate, 1 mM pyruvate, 20 U/mL penicillin G, 20 µg/mL streptomycin sulfate, supplemented with 10% FCS) in wells of 24-well dishes. The cells were incubated in the humidified atmosphere of a CO_2_ incubator (Sanyo, Osaka, Japan) containing 10% CO_2_. The culture medium was renewed every seventh day and one day prior to an experiment. For experiments, confluent primary astrocyte cultures of an age between 14 and 28 days after seeding were used. Astrocyte-rich primary cultures are strongly enriched in astrocytes and contain only low numbers of contaminating other types of glial cells [33–35].

### Experimental Incubation of Cultured Astrocytes

If not stated otherwise, astrocyte cultures in wells of 24-well dishes were washed twice with 1 mL pre-warmed (37 °C) glucose-free incubation buffer (IB; 145 mM NaCl, 20 mM HEPES, 5.4 mM KCl, 1.8 mM CaCl_2_, 1 mM MgCl_2_, 0.8 mM Na_2_HPO_4_, pH adjusted with NaOH to 7.4 at 37 °C). Subsequently, the cells were incubated for 60 min in 250 µL IB with 1 µM of the uncoupler BAM15 at 37 °C in the humidified atmosphere of a CO_2_-free incubator to deprive the cells of ATP. To study ATP restoration after the BAM15 treatment, the cells were washed twice with 1 mL pre-warmed (37 °C) glucose-free IB to remove the uncoupler and incubated in 250 µL IB containing substrates and/or inhibitors as indicated in the legends of the figures and tables for up to 6 h in the humidified atmosphere of a CO_2_-free incubator (37 °C). After the given incubation periods, the incubation media were harvested to test for potential toxicity of the incubation conditions by measuring the activity of extracellular LDH and to determine lactate contents. The cells were washed twice with 1 mL ice-cold (4 °C) phosphate-buffered saline (PBS; 10 mM potassium phosphate buffer pH 7.4 containing 150 mM NaCl) and lysed for quantification of ATP and other cellular metabolites.

### Determination of Cellular Contents of ATP, ADP and AMP

The cellular contents of ATP, ADP and AMP were determined in perchloric acid lysates of cultured astrocytes as previously described in detail [11, 14]. Briefly, the washed cultures were lysed in 400 µL of ice-cold 0.5 M HClO_4_ on ice for 5 min. The lysates were collected and neutralized by adding 2 M KOH. ATP in the lysate was determined as recently described [11] by a luciferin-luciferase-based luminometric assay using the Cell Titer Glo^®^ 2.0 ATP Assay Kit. The ADP and AMP present in the lysates were converted by enzymatic reactions to ATP as recently described in detail [14] that was also quantified by the luminometric ATP assay. The specific cellular contents of the three adenosine phosphates were calculated by normalizing the determined cellular contents to the respective initial cellular protein contents of the cultures.

### Determination of Cellular Contents of Nicotinamide Coenzymes

The cellular contents of NAD^+^, NADH, NADP^+^ and NADPH were determined in alkaline lysates [36, 37] by modified protocols of reported enzymatic cycling assays [17]. Control experiments revealed that the NAD(H) (sum of NAD^+^ plus NADH) cycling assay used is specific for the detection of NAD^+^ and NADH and does not give any signals in the presence of NADP^+^ or NADPH, while the NADP(H) (sum of NADP^+^ plus NADPH) cycling assay used is specific for NADP^+^ and NADPH and does not give any signals in the presence of NAD^+^ or NADH (data not shown).

After a given treatment, cultured astrocytes were lysed for the quantification of the four nicotinamide coenzymes in 500 µL alkaline extraction buffer (20 mM NaHCO_3_, 100 mM Na_2_CO_3_, 15 mM nicotinamide, 0.05% Triton X-100, pH 11) for 10 min on ice in the dark. The lysed cells from two wells were pooled and homogenized in an ice-cooled ultrasonication water bath with 35 kHz (Sonorex, Bandelin, Berlin, Germany) for 5 min. Subsequently, 250 µL of the homogenized lysate was heated for 30 min at 60 °C to completely destroy the oxidized coenzymes NAD^+^ and NADP^+^, while the reduced coenzymes remain intact [37].

For determination of NAD(H), 50 µL of the heated alkaline lysates (for the quantification of NADH), of the not heated alkaline lysates (for the quantification of NADH plus NAD^+^) or of NADH standards (0-100 pmol/50 µL alkaline extraction buffer) were diluted in wells of a microtiter plate with 50 µL extraction buffer and 100 µL reaction mixture (300 mM Tris-HCl buffer pH 8.0, containing 12 mM EDTA, 0.6 mM MTT, 3 mM PMS and 45 U/mL LDH). The cycling reaction was started by the addition of 100 µL 0.5 M lactate (in water) to each well. The increase in absorbance of the MTT formazan generated was monitored at 570 nm for 10 min using the Multiskan Sky Microplate Spectrophotometer (Thermo Fisher Scientific, Bremen, Germany). The calibration curve created from the slopes of the linear increases in the absorbance over time recorded for the standards was used to calculate the amount of total cellular NAD(H) and of NADH in the non-heated and heated lysates, respectively. The cellular NAD^+^ content was calculated by subtracting the cellular NADH content (heated lysate) from the total cellular NAD(H) content (not heated lysate). The specific NAD(H) content was calculated by normalising the cellular NAD(H) content per well to the initial cellular protein content of the respective culture.

To quantify the cellular NADP(H) (sum of NADP^+^ and NADPH) content, 100 µL of the heated alkaline lysate (for the quantification of NADPH), of the not heated lysates (for the quantification of NADPH plus NADP^+^) or of NADPH standards (0–60 pmol/100 µL alkaline extraction buffer) were diluted with 100 µL reaction mixture (300 mM Tris-HCl buffer pH 8.0, 15 mM EDTA, 1.5 mM MTT, 3 mM PES, 30 U/mL glucose-6-phosphate dehydrogenase). The cycling reaction was started by the addition of 100 µL 3 mM glucose-6-phosphate (in water) to each well. The increase in absorbance of the MTT formazan generated was monitored at 570 nm for 10 min. NADP(H) was quantified by making use of the signals observed for NADPH standards as described above for the NAD(H) quantification.

### Determination of Extracellular Lactate

The concentration of extracellular lactate that was produced and released from the cells during a given incubation was determined by a coupled enzymatic assay with LDH and glutamate-pyruvate transaminase in an alkaline glutamate buffer in microtiter plate format as previously described [33]. The NADH generated from lactate in the assay was quantified by determining the increase in absorption at 340 nm and this value was used to calculate the extracellular concentration of lactate [33].

### Determination of Cellular Protein Content and Cell Viability

The initial cellular protein content per well was determined by the Lowry method [38] using bovine serum albumin as standard protein. A potential loss of cell viability by a given treatment was tested for by measuring the extracellular activity of LDH (in 10 µL harvested media samples after a given incubation) and comparing this activity with the initial cellular LDH activity determined for Triton X-100 lysates (100% cellular LDH activity) of untreated cells. The extracellular LDH activity found after a given treatment was compared to the initial cellular LDH activity as previously described [33].

### Data Presentation and Statistical Analysis

The data shown in figures and tables represent means ± standard deviations (SDs) of values obtained from n independent experiments that had been performed in duplicates on the given number of independently prepared astrocyte cultures. For data sets from less than 5 independent experiments, statistical analysis was done under the assumption of normal distribution. Analysis for statistical significance between groups of data was done by ANOVA (followed by the Bonferroni post-hoc test). Experiments showing time-dependencies of various treatments were analyzed by a two-way ANOVA for the factors treatment and incubation time. Differences between two groups of data were tested for statistical significance by the paired t-test. The calculated levels of significance compared to the respective control conditions are given in the figures and tables as described in the respective legends. *p* > 0.05 was considered as not significant.

## Results

### Depletion of the Cellular ATP Content of Cultured Astrocytes by Glucose Deprivation and BAM15 Application

To study ATP restoration of cultured astrocytes, the initial high cellular ATP content of such cultures was first lowered by a preincubation with the uncoupler BAM15 in glucose-free buffer as previously described [12, 18]. Data obtained in a total of 30 experiments revealed that a 60 min incubation with 1 µM BAM15 lowered the initial cellular ATP content from an initial value of 32.2 ± 4.7 nmol/mg by 80% to 6.7 ± 2.7 nmol/mg, while no significant loss in cellular ATP content was observed for glucose-deprived astrocytes that had been incubation in the absence of the uncoupler (Tables 1 and 2). In contrast to the cellular ATP level, a 60 min glucose-free incubation without or with BAM15 did not cause large differences in the specific cellular contents of the nicotinamide coenzymes NAD^+^, NADH, NADP^+^ and NADPH nor in the percental ratios of NADH/NAD(H) or NADPH/NADP(H). However, compared to the initial values some decline in the cellular contents of nicotinamide coenzymes was found for both incubation conditions (Table 2) which is consistent with literature data and most likely caused by the absence of NAD^+^ precursors during the incubation [39]. None of the conditions applied caused any obvious damage of the cells as indicated by the absence of any substantial increase in extracellular LDH activity (Tables 1 and 2).

**Table 1:** ATP content and cell viability of BAM15-treated cultured astrocytes

Treatment	Incubation time (min)	ATP content		LDH release (%)
		(nmol/mg)	(% of initial)	
None	0	32.2 ± 4.7	100	0
Without BAM15	60	31.0 ± 4.9	97 ± 13	5 ± 4
With BAM15	60	6.7 ± 2.7^###^	21 ± 9^###^	4 ± 1

The cells were incubated for 60 min in the absence or the presence of 1 µM of the uncoupler BAM15 before the cellular ATP content and the extracellular LDH activity (as indicator for a potential cell toxicity, given as % of total cellular LDH activity) were determined. The data presented are means ± SD of values that were obtained in 30 (ATP) and 13 (LDH) experiments on 25 (ATP) and 13 (LDH) independently prepared cultures. The significance of differences (paired t-test) between data for incubations without and with BAM15 is indicated by ^###^*p* < 0.001

**Table 2:** Metabolic parameters of BAM15-treated cultured astrocytes

Incubation time	0 min	60 min	60 min
Presence of BAM15	no	no	yes
ATP content (nmol/mg)	29.2 ± 1.8	29.6 ± 4.1	6.0 ± 4.5***^, ###^
NAD(H) content (nmol/mg)	2.74 ± 0.14	1.97 ± 0.20**	1.91 ± 0.33**
NAD^+^ content (nmol/mg)	1.66 ± 0.07	1.49 ± 0.16	1.54 ± 0.25
NADH content (nmol/mg)	1.08 ± 0.16	0.48 ± 0.20*	0.37 ± 0.18**^, ##^
NADH content (% of NAD(H))	39 ± 4	24 ± 8	19 ± 7*^, #^
NADP(H) content (nmol/mg)	0.61 ± 0.03	0.52 ± 0.05	0.47 ± 0.03**
NADP^+^ content (nmol/mg)	0.34 ± 0.14	0.32 ± 0.06	0.35 ± 0.03
NADPH content (nmol/mg)	0.27 ± 0.14	0.20 ± 0.07	0.12 ± 0.05^#^
NADPH content (% of NADP(H))	44 ± 22	38 ± 12	26 ± 10^##^
Extracellular LDH activity (%)	0	2 ± 4	4 ± 2

The cells were incubated without glucose for 60 min in the absence or the presence of 1 µM of the uncoupler BAM15. The given cellular metabolic parameters of the cultures were determined before (0 min, untreated cells) and after the 60 min incubation. NAD(H) represents the sum of NAD^+^ plus NADH, while NADP(H) represents the sum of NADP^+^ plus NADPH. The extracellular LDH activity (given as % of the initial cellular LDH activity) was measured after the 60 min incubation as indicator for potential cell toxicity. The data presented are means ± SD of values that were obtained in experiments on three independently prepared cultures. The initial cellular LDH activity was 192 ± 20 nmol/(min x well) and the initial cellular protein content was 158 ± 11 µg/well. The significance of differences (ANOVA) compared to the initial values of untreated cells is indicated by asterisks (*p < 0.05, **p < 0.01 and ***p < 0.001) and that between data for incubations without and with BAM15 (paired t-test) is indicated by hashes (^#^*p* < 0.05, ^##^*p* < 0.01 and ^###^*p* < 0.001)

### Restoration of the Cellular ATP Content of Astrocytes in the Presence of Energy Substrates and/or Adenosine

**Fig. 1:**
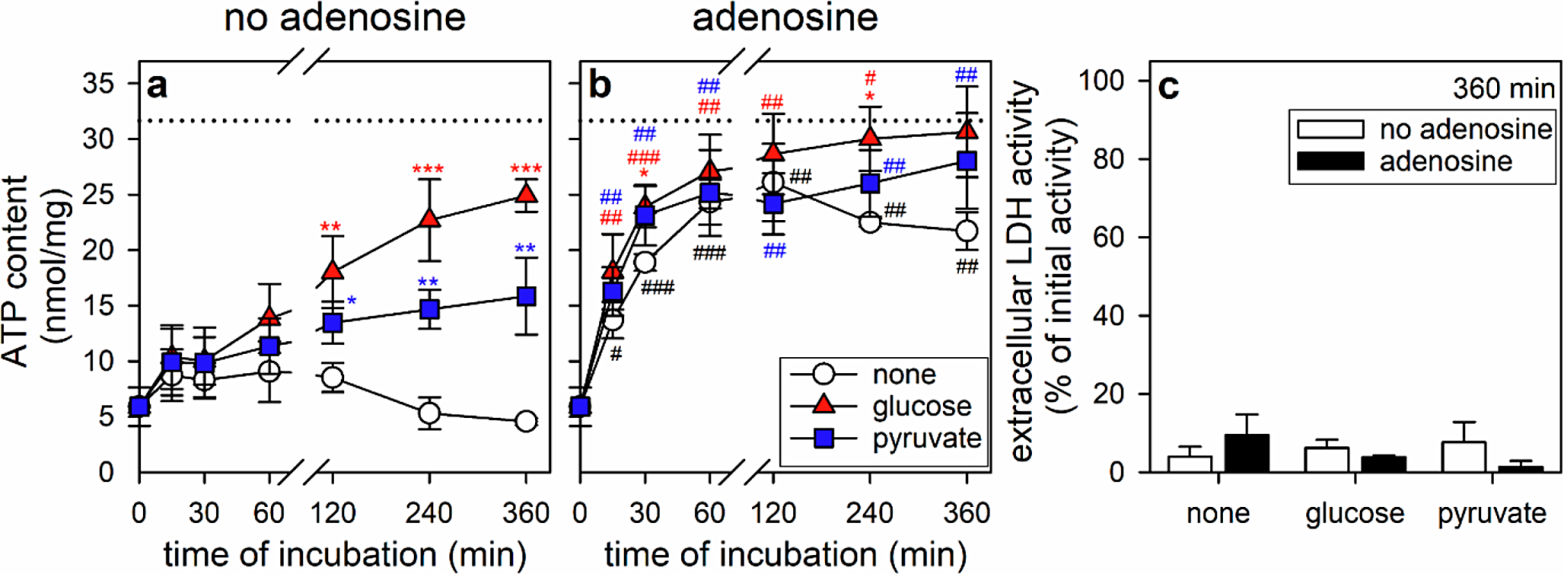
Time-dependency of the ATP restoration by ATP-deprived cultured astrocytes. The cells had been preincubated for 60 min in glucose-free IB with 1 µM of BAM15 to lower the cellular ATP content before the cells were incubated without (none) or with 5 mM glucose or 5 mM pyruvate in the absence (**a**, **c**) or the presence (**b**, **c**) of 1 mM adenosine. The cellular ATP content was determined for the given incubation periods (**a**, **b**) and the extracellular LDH activity for the 360 min incubation (**c**). The data shown are means ± SD of values obtained in three experiments performed on independently prepared cultures. The average initial ATP content of the cultures was 31.7 ± 0.6 nmol/mg as indicated by the black dotted lines in panels a and b. The initial cellular LDH activity was 103 ± 5 nmol/(min x well) and the initial cellular protein content was 113 ± 1 µg/well. The significance of differences (ANOVA) of data compared to the respective glucose- and pyruvate-free control condition (none) is indicated by *p < 0.05, **p < 0.01 and ***p < 0.001 in the colors of the respective conditions. The significance of differences (paired t-test) between data for incubations without (**a**) and with (**b**) adenosine are indicated by hashes in panel b with ^#^*p* < 0.05, ^##^*p* < 0.01 and ^###^*p* < 0.001. Two-way ANOVA revealed that the time of the main incubation (no adenosine: F = 5.27, *p* < 0.001; adenosine: F = 67.49, *p* < 0.001) as well as the kind of treatment (no adenosine: F = 17.97, *p* < 0.001; adenosine: F = 12.9, *p* < 0.001) have highly significant effects on the ATP restoration

**Fig. 2:**
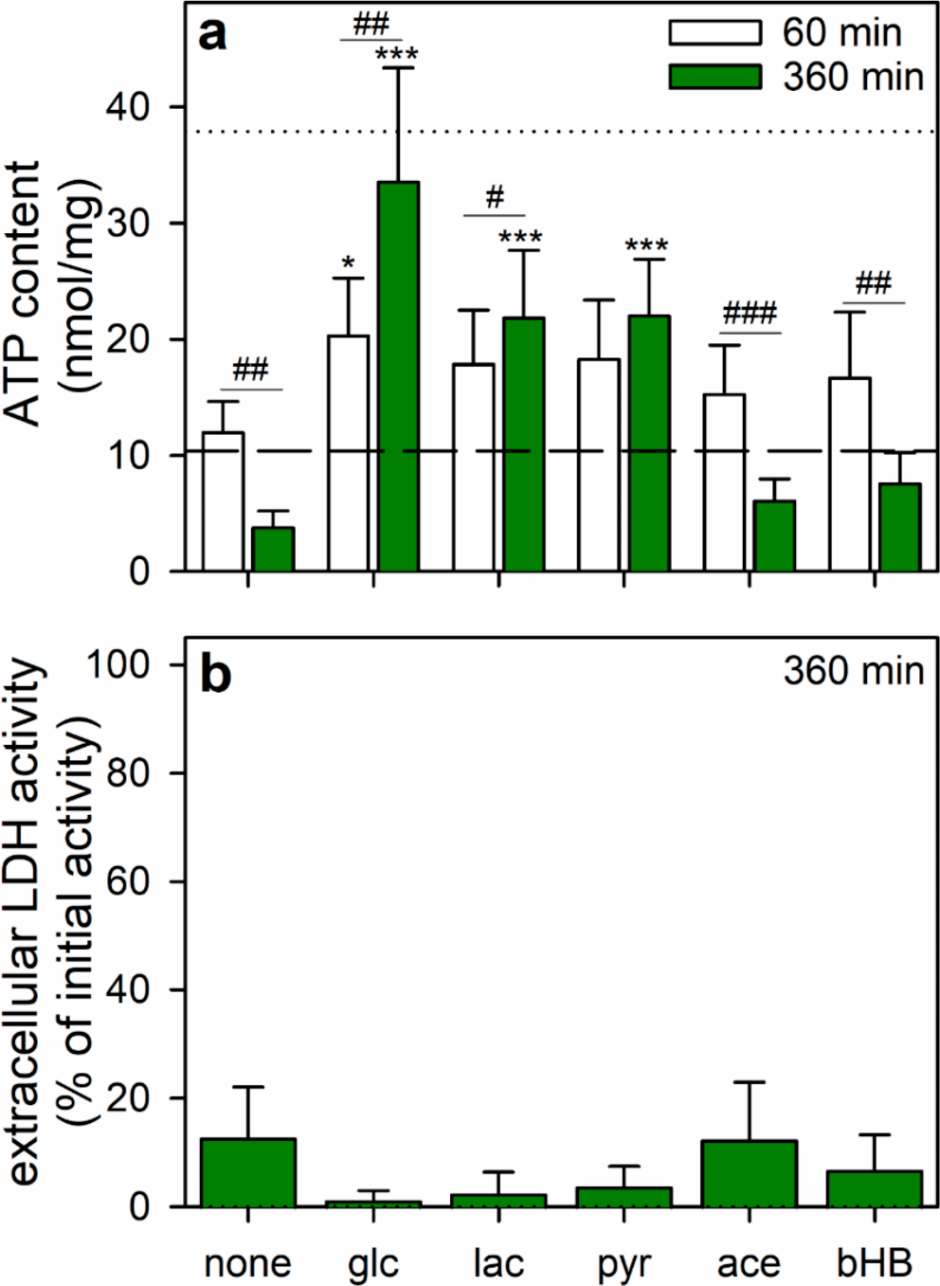
Use of various energy substrates for ATP restoration by ATP-deprived astrocytes. Astrocyte cultures had been preincubated for 60 min in glucose-free IB with 1 µM of BAM15 to lower the cellular ATP content before the cells were incubated in glucose-free IB that had been supplemented with 5 mM of the given energy substrates. After 60 or360 min, the cellular ATP content (**a**) and after 360 min the extracellular LDH activity (**b**) were determined. The data shown are means ± SD of values obtained in six experiments performed on independently prepared cultures. The average initial ATP content of the cultures (37.9 ± 5.2 nmol/mg) is indicated by the black dotted line in panel a and the ATP content determined for the 60 min BAM15 preincubation (10.4 ± 2.6 nmol/mg) is indicated by the black dashed line in panel a. The cultures contained 144 ± 21 µg protein/well and an initial cellular LDH activity of 177 ± 62 nmol/(min × well). The significance of differences (ANOVA) compared with the data obtained for the respective control incubation (none, absence of any exogenous substrates) is indicated by *p < 0.05 and ***p < 0.001. The significance of differences (t-test) between the data obtained for the 1 h and 6 h incubation of a given condition is indicated by ^#^*p* < 0.05, ^##^*p* < 0.01 and ^###^*p* < 0.001. glc, glucose; lac, lactate; pyr, pyruvate; ace, acetate; bHB, β-hydroxybutyrate

To test for the potential of BAM15-treated ATP-depleted astrocytes to restore their cellular ATP content after removal of the uncoupler, the cells were washed and incubated in amino acid-free IB in the absence or the presence of 5 mM glucose. During a 6 h incubation in glucose-free IB, the low ATP content determined for BAM15-treated astrocytes (around 25% of the initial cellular ATP content) remained stable for up to 2 h (Fig. 1a; Table 3), but decreased further to around 10% of the initial ATP content during longer incubations for up to 6 h (Fig. 1a; Table 3). In contrast, the cellular ATP content of glucose-fed cells increased during incubations for 60 min and 6 h to amounts that accounted for around 50% and 80%, respectively, of the initial cellular ATP content of untreated cells (Fig. 1a; Table 3). If glucose was substituted by pyruvate, the cellular ATP restoration was less efficient although the cellular ATP content was found significantly increased and around half of the cellular ATP that had been lost during the BAM15 treatment was restored within 6 h (Figs. 1a and 2a). Partial ATP restoration by ATP-depleted astrocytes was also observed for a treatment with lactate, but not for 6 h incubations with acetate or β-hydroxybutyrate (Fig. 2a).

**Table 3:** ATP restoration after application of glucose and/or adenosine in BAM15-treated cultured astrocytes

Condition	ATP content		Number of experiments (independent cultures)
	(nmol/mg)	(% of initial)	
Untreated cells	32.8 ± 6.0	100	17 (13)
BAM15-preincubated cells	7.6 ± 3.3	23 ± 9	17 (13)
**60 min main incubation**			
No substrate	10.0 ± 3.3***	30 ± 8***	15 (12)
Glucose	16.4 ± 5.6***^, ##^	49 ± 13***^, #^	14 (13)
Adenosine	25.7 ± 6.9*^, ###^	78 ± 13***^, ###^	17 (13)
Glucose + adenosine	31.4 ± 9.0 ^###^	94 ± 13 ^###^	13 (12)
**360 min main incubation**			
No substrate	3.3 ± 1.7***	8 ± 5***	12 (12)
Glucose	27.6 ± 9.0 ^###^	80 ± 11***^, ###^	12 (12)
Adenosine	17.6 ± 4.8***^, ###^	54 ± 15***^, #^	12 (12)
Glucose + adenosine	34.0 ± 14.1^###^	102 ± 19 ^###^	9 (9)

The cells had been preincubated for 60 min in the presence of 1 µM of the uncoupler BAM15 to deplete astrocytes of ATP and were subsequently exposed for 60 or360 min in the absence or the presence of 5 mM glucose and/or 1 mM adenosine before the cellular ATP content was determined. The data presented are means ± SD of values that were obtained in n experiments on the given number (in brackets) of independently prepared cultures. The significance of differences (ANOVA) compared to the initial ATP content (given as asterisks) and to the ATP content determined after the BAM15 treatment (given as hashes) are indicated by *^, #^*p* < 0.05, ^##^*p* < 0.01 and ***^, ###^*p* < 0.001. Two-way ANOVA revealed that the time of the main incubation (F = 54.91, *p* < 0.001) and the kind of treatment (F = 27.33, *p* < 0.001) have highly significant effects on the ATP restoration

The slow and partial ATP restoration observed in ATP-deprived astrocytes after application of glucose (Fig. 1a) was strongly accelerated by coapplication of 1 mM adenosine as precursor for the purine moiety of ATP (Fig. 1b; Table 3) and the initial cellular ATP content was found fully reestablished already within 60 min (Fig. 1b; Table 3). However, also the application of adenosine alone in glucose-free IB enabled BAM15-preincubated astrocytes to restore within 60 min around 80% of their initial ATP content (Fig. 1b; Table 3). This adenosine-mediated increase was transient and the cellular ATP content was found lowered after longer incubations for 6 h in glucose-free IB (Fig. 1b; Table 3). However, the decline in ATP content of adenosine-treated cells during longer incubations was prevented by the presence of glucose or pyruvate as energy substrates (Fig. 1b; Table 3). None of the conditions applied caused any obvious cell toxicity as demonstrated by the low extracellular LDH activity found after incubations for 360 min (Figs. 1c and 2b).

### Effects of a BAM15-Treatment and -Removal on the Cellular Contents of ATP, ADP and AMP in Astrocytes

**Fig. 3:**
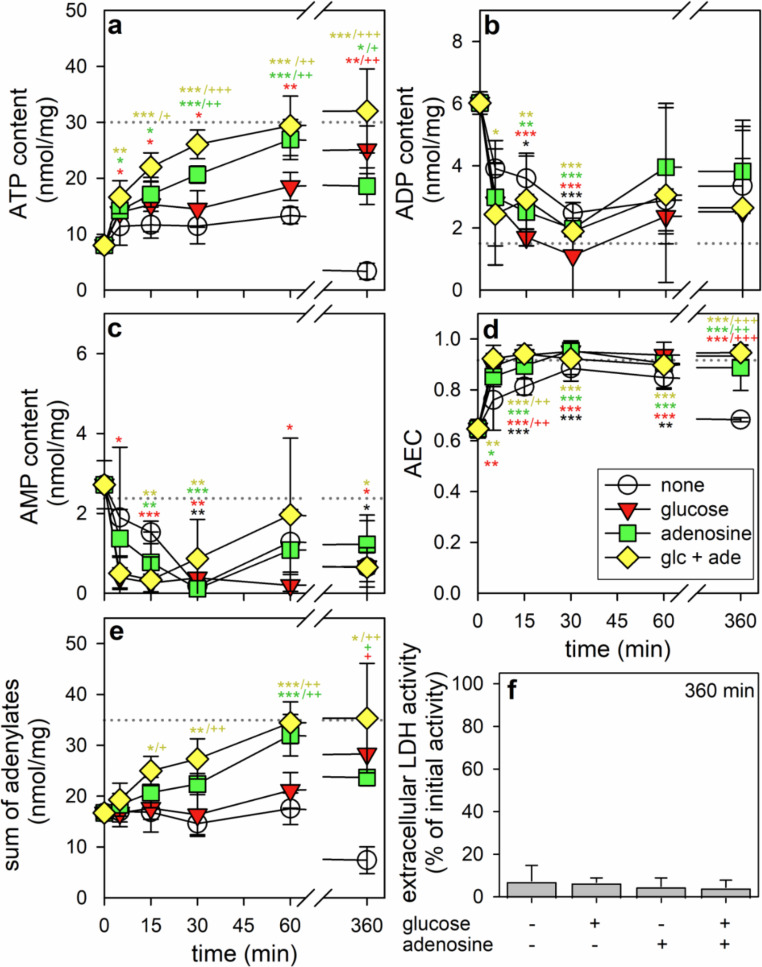
Alterations in cellular adenosine phosphate contents during ATP restoration in ATP-deprived cultured astrocytes. The cells had been preincubated for 60 min in glucose-free IB with 1 µM of BAM15 before the cells were incubated without (none) or with 5 mM glucose and/or 1 mM adenosine for up to 360 min. The cellular specific contents of ATP (**a**), ADP (**b**) and AMP (**c**) were determined for the given incubation periods and the extracellular LDH activity for the 360 min incubation (**f**). In addition, the AEC (**d**) and the sum of adenosine phosphates (**e**) were calculated. The data shown are means ± SD of values obtained in three experiments performed on independently prepared cultures. The average initial values determined for untreated cultures (30.1 ± 6.5 nmol ATP/mg; 1.5 ± 0.8 nmol ADP/mg; 2.4 ± 2.7 nmol AMP/mg; AEC: 0.92 ± 0.04; sum of adenosine phosphates: 35.2 ± 6.6 nmol/mg) are indicated by the black dotted lines. The initial cellular LDH activity was 110 ± 28 nmol/(min x well) and the initial cellular protein content was 99 ± 23 µg/well. The significance of differences (ANOVA) of data compared to the values determined after the 60 min preincubation (0 min on x-axis) is indicated by *p < 0.05, **p < 0.01 and ***p < 0.001 in the colors of the respective conditions. The significance of differences (ANOVA) of data compared to the control condition (none) is indicated by ^+^*p* < 0.05, ^++^*p* < 0.01 and ^+++^*p* < 0.001 in the colors of the respective conditions. Two way ANOVA revealed that the time of the main incubation (ATP: F = 12.69, *p* < 0.001; AEC: F = 37.14, *p* < 0.001; sum of adenylates: F = 5.58, *p* < 0.001) as well as the kind of treatment (ATP: F = 20.63, *p* < 0.001; AEC: F = 14.26, *p* < 0.001; sum of adenylates: F = 14.68, *p* < 0.001) have highly significant effects on the ATP restoration, the AEC and the sum of adenylates. The cellular contents of ADP and AMP were significantly affected by the time of main incubation (ADP: F = 14.78, *p* < 0.001; AMP: F = 12.81, *p* < 0.001), but not by the kind of treatment (ADP: F = 1.99, *p* = 0.125; AMP: F = 1.9, *p* = 0.139). glc, glucose; ade, adenosine

During the 60 min incubation of astrocytes in glucose-free IB with BAM15, the cellular ATP content had declined from 30.1 ± 6.5 nmol/mg to 8.0 ± 7.2 nmol/mg, while the cellular contents of ADP and AMP were found increased from 1.5 ± 0.8 nmol/mg to 6.0 ± 0.4 nmol/mg and unaltered (2.4 ± 2.7 nmol/mg and 2.7 ± 0.6 nmol/mg), respectively (Fig. 3). In addition, the sum of the three adenosine phosphates had declined after the BAM15-treatment from 35.2 ± 6.6 nmol/mg to 16.7 ± 1.2 nmol/mg and the AEC from 0.92 ± 0.04 to 0.65 ± 0.04 (Fig. 3). Removal of the uncoupler caused for all incubation conditions a rapid initial increase in ATP content by up to 8 nmol/mg (Fig. 3a) that was accompanied by a decline in the cellular contents of ADP and AMP (Fig. 3b, c), which is likely to be the consequence of rapid phosphorylation of ADP and AMP to ATP after application of substrates. Although this process did hardly affect the sum of adenosine phosphates (Fig. 3e), the high AEC was almost fully reestablished within 5 min of incubation in the presence of substrates (glucose and/or adenosine) and also within 30 min in substrate-free buffer (Fig. 3d). During longer incubations, the cellular ATP contents were restored (Fig. 3a) to the extents previously determined for the different incubation conditions applied (Fig. 1; Table 3), while the specific cellular contents of ADP (Fig. 3b) and AMP (Fig. 3c) as well as the AEC (Fig. 3d) did not differ anymore from the respective values of untreated cells. Only for BAM15-pretreated astrocytes that had been incubated without substrates for 6 h the ATP content (Fig. 3a) and the sum of adenosine phosphates (Fig. 3e) were found lowered compared to the data found for cells directly after the BAM15-exposure and the calculated AEC (Fig. 3d) was similarly low for this condition. None of the conditions applied caused any obvious cell toxicity as demonstrated by the low extracellular LDH activity (Fig. 3f).

### Test for the Involvement of Mitochondrial Processes in the ATP Restoration by ATP-Deprived Astrocytes

To test for a potential contribution of mitochondrial metabolisms in ATP restoration during incubations with adenosine and/or glucose, BAM15-treated astrocytes were incubated for 60 min without or with 5 mM glucose and/or 1 mM adenosine in the absence or the presence of UK5099, an inhibitor of the mitochondrial pyruvate carrier [40–42], antimycin A, an inhibitor of complex III of the respiratory chain [12, 14, 43], or with the uncoupler BAM15 [12, 18, 44, 45]. An adenosine concentration of 1 mM was chosen for this type of experiment to enable the cells to produce in the absence of glucose reasonable amounts of lactate from the nucleoside as exclusive exogenous substrate.

**Fig. 4:**
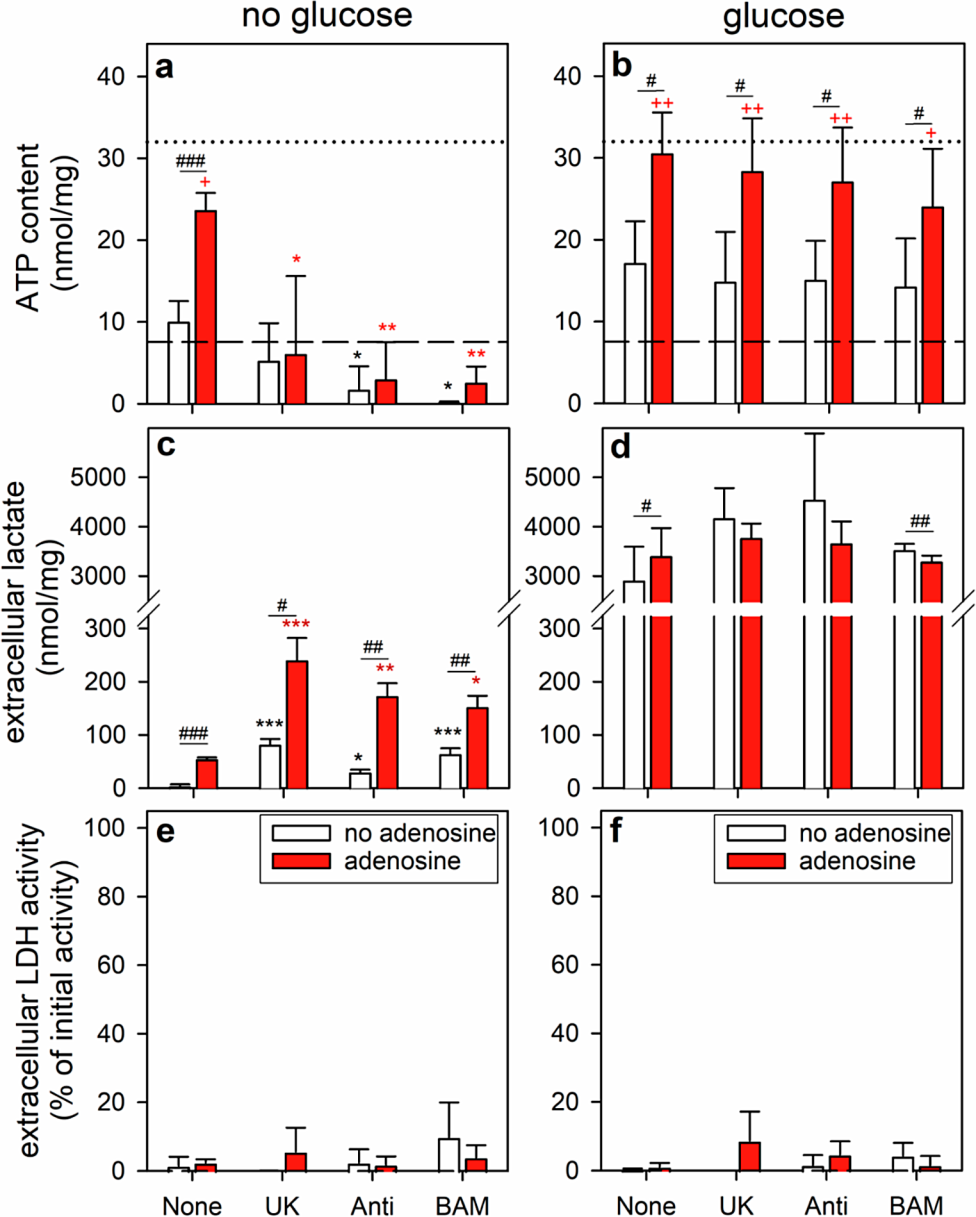
Effects of mitochondrial inhibitors on ATP restoration and lactate release by ATP-deprived astrocytes. The cultures had been preincubated for 60 min in glucose-free IB with 1 µM of BAM15 to lower the cellular ATP content before the cells were incubated in the absence (**a**, **c**, **e**) or the presence (**b**, **d**, **f**) of 5 mM glucose without or with 1 mM adenosine. For these conditions, the cells were coincubated without (none) or with an inhibitor of the mitochondrial pyruvate carrier (UK5099, UK; 10 µM) or of the complex III of the respiratory chain (antimycin A, Anti; 1 µM) or with BAM15 (BAM; 1 µM). After a 60 min main incubation, the cellular ATP content (**a**, **b**), the extracellular lactate accumulation (**c**, **d**) and the extracellular LDH activity (**e**, **f**) were determined. The data shown are means ± SD of values obtained in three experiments performed on independently prepared cultures. The average initial ATP content of the cultures (32.0 ± 5.7 nmol/mg) is indicated by the black dotted lines in panels a and b. The protein content of the cultures was 141 ± 23 µg/well and the initial cellular LDH activity 163 ± 11 nmol/(min × well). The significance of differences (ANOVA) compared with the data obtained for the respective control incubation (none) is indicated by *p < 0.05, **p < 0.01 and ***p < 0.001. The significance of differences (ANOVA) compared with the ATP data obtained for the 60 min preincubation (7.6 ± 3.1 nmol/mg; indicated by the black dashed lines in panels a and b) is indicated by ^+^*p* < 0.05 and ^++^*p* < 0.01. The significance of differences (t-test) between data obtained for adenosine-free and adenosine-containing incubations is indicated by ^#^*p* < 0.05, ^##^*p* < 0.01 and ^###^*p* < 0.001

Compared to the inhibitor-free control condition (none), all three modulators of mitochondrial metabolism prevented the adenosine-induced ATP restoration in the absence of glucose (Fig. 4a), but not in the presence of glucose (Fig. 4b). In addition, the three inhibitors hardly affected the lactate release from glucose-treated astrocytes under the conditions used (Fig. 4d), while the low amounts of lactate released from astrocytes that had been incubated without glucose in the absence or the presence of adenosine were strongly increased by the inhibitors of mitochondrial processes (Fig. 4c). None of the conditions applied caused any obvious damage of the cells as indicated by the absence of any increase in extracellular LDH activity (Fig. 4e, f).

### Concentration Dependency of the Adenosine-Dependent ATP Restoration in ATP-Depleted Astrocytes

**Fig. 5:**
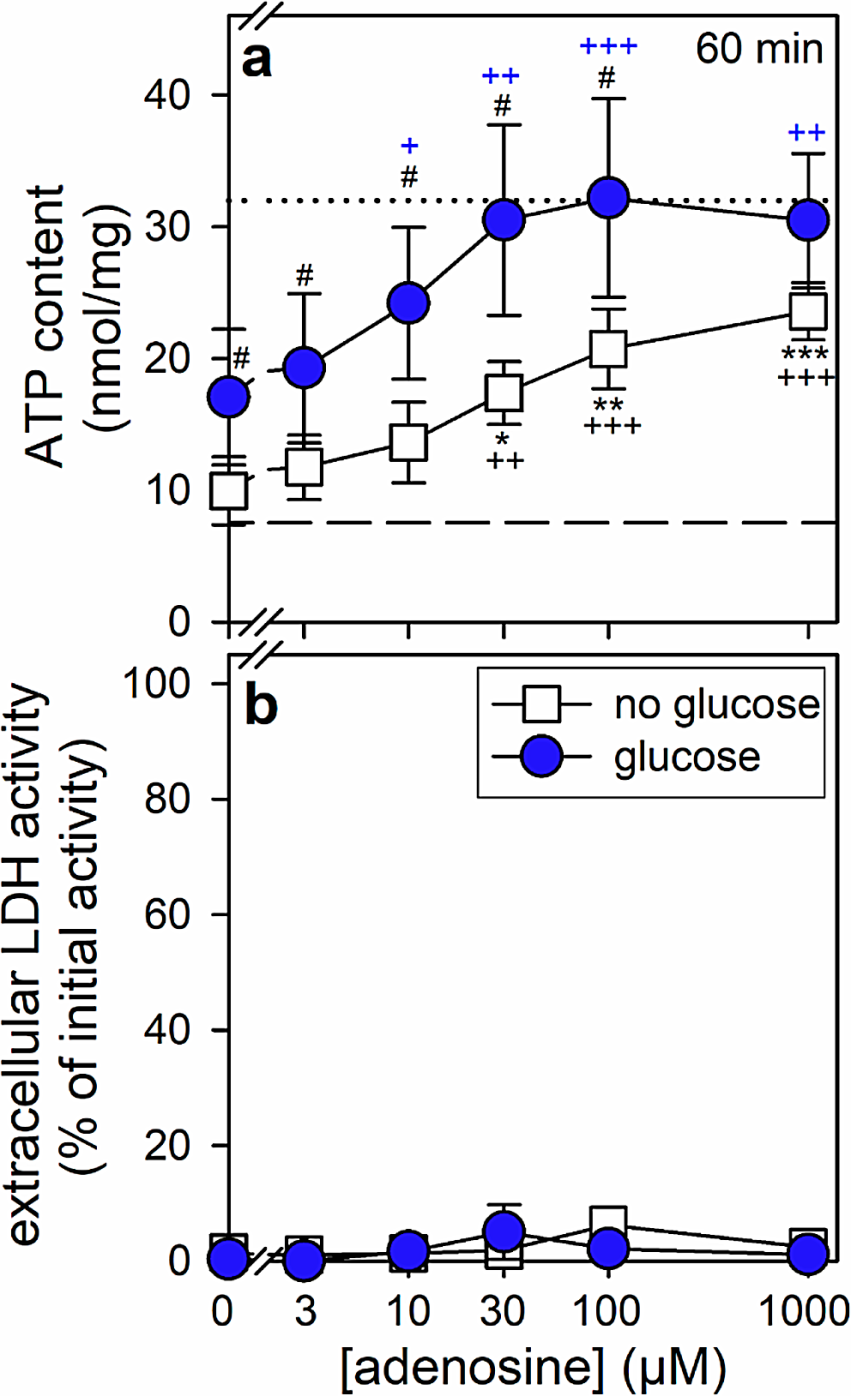
Adenosine dependent ATP restoration by astrocytes. The cells had been pre-incubated for 60 min in glucose-free IB with 1 µM of BAM15 to lower the cellular ATP content before the cells were incubated without or with 5 mM glucose in the presence of the indicated concentrations of adenosine. After 60 min, the cellular ATP content (**a**) and the extracellular LDH activity (**b**) were determined. The data shown are means ± SD of values obtained in three experiments performed on independently prepared cultures. The average initial ATP content of the cultures (32.0 ± 5.7 nmol/mg) is indicated by the black dotted lines in panel a. The cellular protein content was 141 ± 23 µg/well and the initial cellular LDH activity 163 ± 11 nmol/(min × well). The significance of differences (ANOVA) compared with the data obtained for the control incubation (absence of glucose and adenosine) is indicated by *p < 0.05, **p < 0.01 and ***p < 0.001. The significance of differences (ANOVA) compared with the data obtained for the 60 min preincubation (7.6 ± 3.1 nmol/mg; indicated by the black dashed lines in panel a) is indicated by ^+^*p* < 0.05, ^++^*p* < 0.01 and ^+++^*p* < 0.001. The significance of differences (t-test) between data obtained for glucose-free and glucose-containing incubations is indicated by ^#^*p* < 0.05

The presence of adenosine in a concentration of 1 mM allowed ATP-depleted astrocytes to restore within 60 min the cellular ATP content to around 80% in the absence of glucose and to more than 90% in the presence of glucose (Table 3). The observed ATP restoration depended strongly on the concentration of adenosine applied (Fig. 5). In the absence of glucose significant ATP restoration was observed for incubations of BAM15-treated astrocytes with 30 µM adenosine but even higher concentrations of up to 1 mM adenosine did not allow the cells to fully restore their initial ATP content (Fig. 5a). In contrast, much lower concentrations of adenosine were required to efficiently increase the ATP content in glucose-treated BAM15-preincubated astrocytes (Fig. 5a). For such conditions, already adenosine in concentrations of 10 µM and 30 µM allowed half-maximal and maximal ATP restoration (Fig. 5a). For adenosine concentrations of up to 100 µM the coincubation with glucose increased the cellular ATP content determined after the restoration period by around 10 nmol/mg (Fig. 5a). None of the conditions applied caused any obvious cell damage as indicated by the absence of any increase in extracellular LDH activity (Fig. 5b).

### Test for the Consequences of an Impairment of Adenosine Metabolism on the Adenosine-Dependent ATP Restoration by ATP-Deprived Astrocytes

To test how the modulation of cellular adenosine metabolism may affect the ability of adenosine to serve as exogenous substrate for ATP restoration, BAM15-treated astrocytes were incubated in glucose-containing buffer without or with 30 µM adenosine. An adenosine concentration of 30 µM was chosen for this type of experiment as this concentration was found to enabled the cells in the presence of glucose to fully restore the initial ATP content within 60 min (Fig. 5a).

**Fig. 6:**
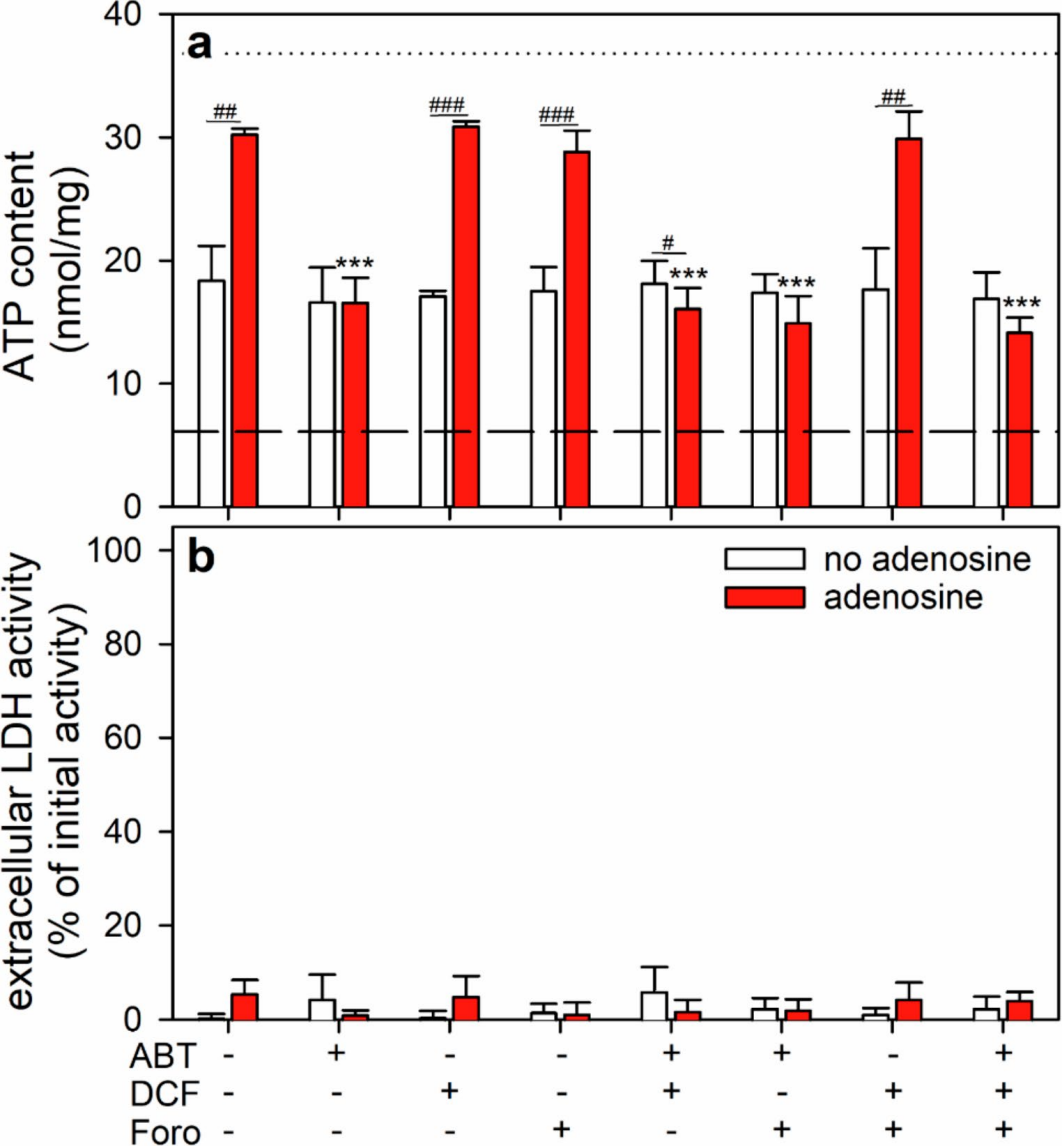
Effects of inhibitors of adenosine metabolism on the utilization of adenosine for ATP restoration in glucose-fed astrocytes. Astrocyte cultures had been preincubated for 60 min in glucose-free IB with 1 µM of BAM15 to lower the cellular ATP content before the cells were incubated in glucose (5 mM)-containing IB without or with 30 µM adenosine in the absence or the presence of the adenosine kinase inhibitor ABT-702 (ABT; 10 µM), the adenosine deaminase inhibitor DCF (1 µM) and/or the purine nucleoside phosphorylase inhibitor forodesine (Foro; 10 µM). After 60 min, the cellular ATP content (**a**) and the extracellular LDH activity (**b**) were determined. The data shown are means ± SD of values obtained in three experiments performed on independently prepared cultures. The average initial ATP content of the cultures (36.8 ± 1.6 nmol/mg) is indicated by the black dotted line in panel a. The protein content of the cultures was 130 ± 12 µg/well and the initial cellular LDH activity 119 ± 5 nmol/(min × well). The significance of differences (ANOVA) compared with the data obtained for the respective control incubation (absence of all inhibitors) is indicated by ***p < 0.001. The significance of differences (t-test) between data for adenosine-free and the respective adenosine-containing incubations is indicated by ^#^*p* < 0.05, ^##^*p* < 0.01and ^###^*p* < 0.001

For those conditions, the cells were able to restore within 60 min 50% (glucose) and 80% (glucose plus adenosine) of their initial ATP content (Fig. 6a). The adenosine-induced acceleration of the ATP restoration was completely prevented by the presence of the adenosine kinase inhibitor ABT-702 (10 µM) [46], while the presence of the adenosine deaminase inhibitor DCF (2’-deoxycoformycin) (1 µM) [47, 48] and/or of the purine nucleoside phosphorylase inhibitor forodesine (10 µM) [49, 50] nor their combinations compromised ATP restoration under the conditions used (Fig. 6a). None of the inhibitors nor their combinations affected the glucose-dependent partial ATP restoration in the absence of adenosine (Fig. 6a) nor the cell viability for any of the conditions investigated (Fig. 6b).

To test how the inhibition of enzymes of the cellular adenosine metabolism may affect the ability of adenosine to serve as exclusive exogenous substrate for ATP restoration, BAM15-treated astrocytes were incubated in glucose-free buffer with 100 µM adenosine. This adenosine concentration was chosen for this type of experiment as the presence of 100 µM of the nucleoside was found to enable the cells to restore in the absence of glucose already half of the initial ATP content within 60 min (Fig. 5a).

**Fig. 7:**
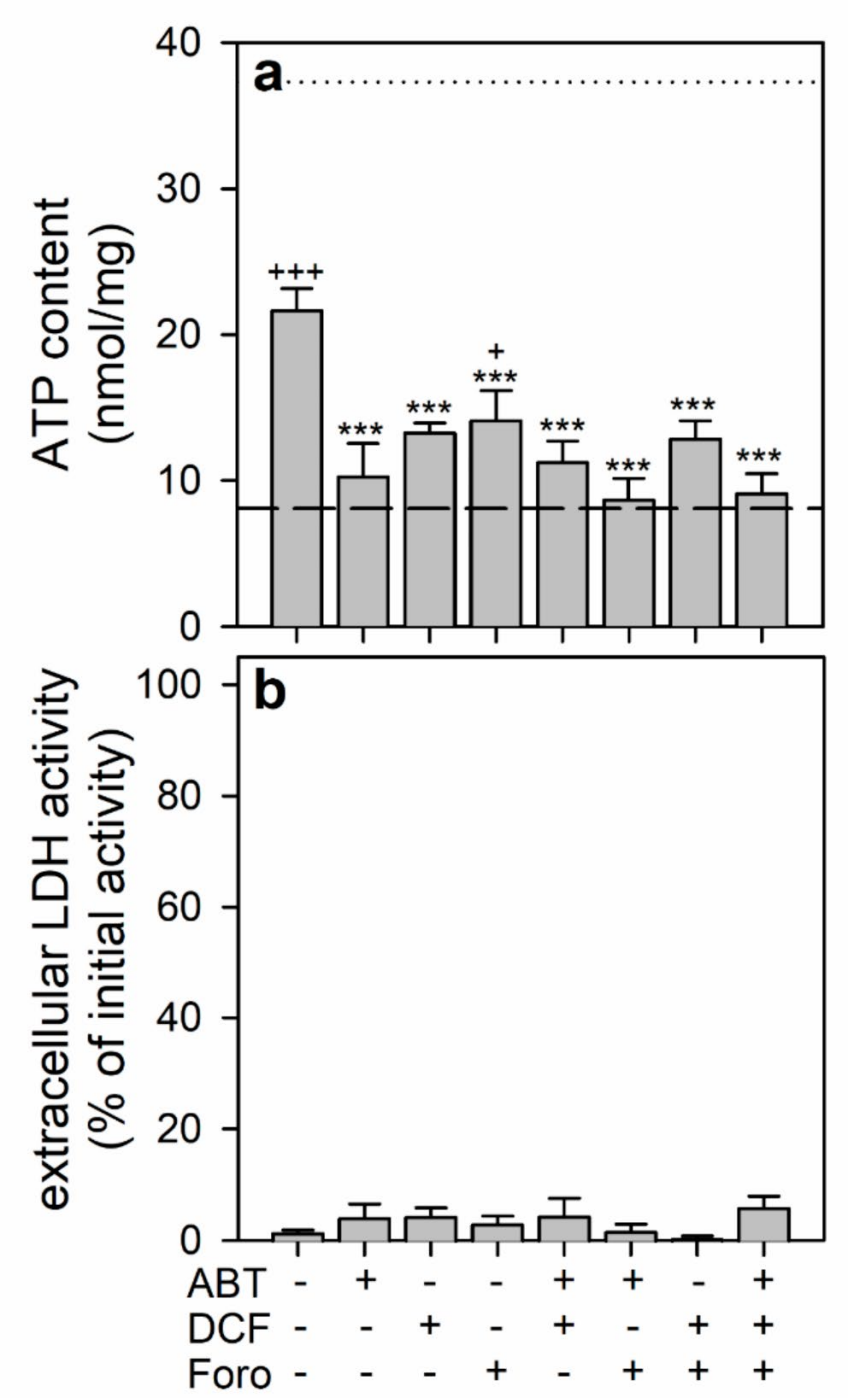
Prevention of the exclusive utilization of adenosine for ATP restoration by inhibitors of adenosine metabolism. Astrocyte cultures had been preincubated for 60 min in glucose-free IB with 1 µM of BAM15 to lower the cellular ATP content before the cells were incubated in glucose-free IB with 100 µM adenosine in the absence or the presence of the adenosine kinase inhibitor ABT-702 (ABT; 10 µM), the adenosine deaminase inhibitor DCF (1 µM) and/or the purine nucleoside phosphorylase inhibitor forodesine (Foro; 10 µM). After 60 min, the cellular ATP content (**a**) and the extracellular LDH activity (**b**) were determined. The data shown are means ± SD of values obtained in three experiments performed on independently prepared cultures. The average initial ATP content of the cultures (37.3 ± 2.4 nmol/mg) is indicated by the black dotted line in panel a. The protein content of the cultures was 136 ± 22 µg/well and the initial cellular LDH activity was 163 ± 59 nmol/(min × well). The significance of differences (ANOVA) compared with the data obtained for the control incubation (absence of all inhibitors) is indicated by ***p < 0.001. The significance of differences (ANOVA) compared with the data obtained after the 60 min pre-incubation (8.1 ± 4.4 nmol/mg; indicated by the black dashed lines in panel a) is indicated by ^+^*p* < 0.05 and ^+++^*p* < 0.001

For those conditions, the cells were able to restore within 60 min around 60% of the initial ATP content (Fig. 7a). This adenosine-dependent ATP restoration was significantly lowered by the presence of the adenosine kinase inhibitor ABT-702, the adenosine deaminase inhibitor DCF or the purine nucleoside phosphorylase inhibitor forodesine, while combinations of the inhibitors had little additive inhibitory effect on the ATP restoration compared to the potential of the single inhibitors to lower ATP restoration (Fig. 7a). None of the inhibitors nor their combinations affected the cell viability for any of the conditions investigated (Fig. 7b).

## Discussion

Cultured astrocytes contain a high cellular ATP content of around 30 nmol/mg that corresponds to a cytosolic ATP concentration of around 7 mM [12]. This high ATP content is maintained in astrocytes by glycolytic and mitochondrial ATP regeneration from ADP [12–14, 18] by making use of the energy obtained during the catabolism of glucose and other substrates [12, 13]. In order to study ATP restoration, cultured astrocytes were first depleted of a substantial part of their cellular ATP by a 60 min preincubation with the mitochondrial uncoupler BAM15 in the absence of glucose. Such a treatment lowered the cellular ATP content within 60 min by around 75% without compromising cell viability, consistent with recent literature data [12, 13]. Compared with this BAM15 treatment, other inhibitors of mitochondrial oxidative phosphorylation such as rotenone, antimycin A or oligomycin are known to deplete cultured astrocytes of ATP even faster and more efficiently [18]. Reason for the lower potential of BAM15 to rapidly deplete cultured astrocytes of ATP compared to inhibitors of respiratory chain complexes [12, 18] may be a residual ADP phosphorylation by ATP synthase in the presence of a rather low proton gradient that may be established by mitochondrial respiration even in the presence of the uncoupler.

The BAM15 exposure was chosen as experimental paradigm as this treatment did not completely deplete astrocytes of their ATP leaving still a millimolar concentration of ATP in the cells which should be sufficient to enable rapid phosphorylation of substrates that will be applied to fuel ATP restoration. In addition, as the BAM15-treatment did not severely affect cellular levels or ratios of the nicotinamide coenzymes, a potential impairment of important oxidoreductase reactions has not to be considered for the restoration period. Finally, the observed rapid and complete restoration of ATP in BAM15-exposed astrocytes in the presence of suitable mitochondrial substrates demonstrates that the uncoupler was efficiently removed by the washing steps to allow ATP restoration. This was not the case for astrocytes that had been deprived of ATP by treatments with the complex I inhibitor rotenone [51, 52], the complex III inhibitor antimycin A [43] or the ATP synthase inhibitor oligomycin [53, 54] (data not shown), most likely due to insufficient removal of the inhibitors by the washing steps performed before the restoration medium was applied.

The observed loss in the cellular ATP content of BAM15-treated astrocytes was accompanied by a small increase in the cellular levels of ADP and AMP. Similar data have recently been reported for astrocytes that had been incubated with antimycin A either in the absence of glucose or in the presence of 2-deoxyglucose [14]. A rapid cellular accumulation of ADP in starved and BAM15-treated astrocytes was expected as consequence of an impaired ADP phosphorylation by inhibition of both glycolysis and oxidative phosphorylation. As a further consequence the accumulated ADP will be used as substrate of adenylate kinase [55], which phosphorylates one ADP molecule by phosphate transfer from a second ADP molecule [56], thereby causing the increase in cellular AMP content. The observed increases in ADP and AMP contents as well as the decline in ATP content led to a reduction in the AEC from 0.9 to 0.6, as recently also reported for starved antimycin A-treated astrocytes [14].

The 60 min exposure to BAM15 lowered severely the total amount of the three adenosine phosphates (sum of ATP, ADP and AMP), similar to results reported for starved cultured astrocytes [14]. For brain slices, a similar loss in the total cellular adenosine phosphate pool was observed for various treatments [57, 58] and discussed to be the consequence of metabolic degradation of accumulated cellular AMP by dephosphorylation to adenosine, subsequent deamination to inosine [14, 59, 60] and final break down to non-salvageable purines [61, 62]. The metabolic elimination of AMP may contribute to the regulation of the cellular concentration of accumulated AMP in starved cells [14, 58] and thereby to the regulation of the activation of AMP-mediated signaling processes [63–65].

Removal of BAM15 and subsequent incubation of the cells with glucose caused within 5 min a rapid increase in cellular ATP content by around 8 nmol/mg, which matched the concomitant rapid loss in the cellular contents of AMP and ADP. This suggests that the accumulated ADP and AMP contents in BAM15-treated astrocytes are rapidly phosphorylated within minutes after removal of the uncoupler and application of glucose, thereby reestablishing the high initial AEC of the cells. Glycolytic glucose metabolism is likely to fuel the rapid ADP phosphorylation after glucose application and cytosolic adenylate kinase will phosphorylate AMP to ADP [56, 66]. During longer restoration periods the reestablished low cellular contents of ADP and AMP as well as the high AEC initially found for untreated astrocytes [14, 25–27] were maintained for hours.

After 60 min treatment with BAM15 in the absence of glucose, cultured astrocytes had lost around 50% of the initial sum of all adenosine phosphates which mainly represented loss of ATP. The lowered ATP content found after the BAM15-preincubation was maintained even in the absence of any exogenous substrates for at least 60 min, suggesting that the cells contained after the BAM15-treatment some intracellular substrates that help to prevent a further decline in cellular ATP content during the initial hour of the restoration phase. Most likely mitochondrial ATP regeneration is involved in this maintenance as the already low ATP content declined further in presence of mitochondrial inhibitors such as antimycin A or BAM15. However, the supporting endogenous energy sources appear to be exhausted during longer substrate-free incubations as indicated by the determined low remaining cellular ATP content that accounted for only around 10% of the initial ATP content after 6 h of incubation. Neither this additional starvation period nor any of the many other conditions applied for studying ATP restoration during 6 h incubations following a 60 min ATP depletion by BAM15 in glucose-deprived astrocytes caused any obvious cell toxicity. This is consistent with the reported transient resistance of cultured starved astrocytes against a substantial loss in the cellular ATP content [12, 14, 18, 67].

The presence of glucose during the restoration period enabled BAM15-treated astrocytes to slowly restore their ATP content. Only little ATP was gained by the cells within 60 min after glucose-application, consistent with literature data describing a rather limited ATP restoration for short time incubations after application of glucose and other energy substrates to cultured astrocytes [30]. Nevertheless, after 6 h of incubation in glucose-containing buffer the cells had restored 80% of their initial cellular ATP content. For this ATP restoration new AMP had to be formed to compensate for the loss in cellular adenosine phosphates observed during the BAM15-treatment. Two main pathways may contribute in glucose-fed cultured astrocytes to the synthesis of new AMP that is used as cellular precursor to restore ATP, the purine *de novo* synthesis and the purine salvage pathway [58, 60, 68–70]. *De novo* synthesis of purine bases on a ribose phosphate precursor generates inosine monophosphate (IMP) from amino acid precursors and this IMP is subsequently amidated to AMP [68, 70]. However, as the presence of amino acids does hardly affect ATP restoration during incubations with glucose (data not shown), the *de novo* synthesis of purine nucleoside phosphates appears unlikely to substantially contribute to ATP restoration under the conditions used. This is consistent with the view that *de novo* synthesis of purines is rather low in brain cells, especially after ATP depletion [71–73] and that these cells use mainly the salvage pathway to replenish their purine nucleotide pool [58, 60, 71, 72, 74, 75].

The salvage pathway uses existing purine bases or purine nucleosides to form IMP and AMP [58, 69]. Considering that mainly this salvage pathway is providing the AMP as building unit for the observed ATP restoration in the presence of glucose, it remains to elucidate which cellular purines or purine-containing molecules provide the adenine for the net AMP synthesis that is required for ATP restoration. Potential candidates may be residual purine nucleosides and bases that had been generated from AMP during the ATP depletion phase. Alternatively, degradation of cellular RNA may have provided purine nucleotides during the ATP restoration phase in substrate-restricted medium. At least in starved yeast a transient increase of the cellular levels of nucleosides and purine bases was observed that was connected with RNA degradation and starvation-induced autophagy [76]. Further studies are required to elucidate in more detail the fate of the adenosine phosphates that disappear from cultured astrocytes during starvation in the presence of BAM15 and to identify the endogenous purine-containing compounds that are used by the cells as precursor for the synthesis of new AMP to enable the cells to restore their ATP.

Compared to glucose incubations, a slower partial ATP restoration was observed for astrocytes that had been incubated during the restoration phase with lactate or pyruvate, while no ATP restoration was observe for incubations with acetate or β-hydroxybutyrate. This supports previous studies, which define lactate, pyruvate, acetate and β-hydroxybutyrate as suitable substrates to maintain the cellular ATP level, but not to restore ATP [58, 60, 77–79]. The reason for this finding may be the need for ribose-5-phosphate as precursor for the phosphoribosyl pyrophosphate (PRPP) that has to be available for the synthesis of purine nucleosides via the salvage pathway [60]. As cultured astrocytes are capable to synthesize glucose-6-phosphate from pyruvate and lactate [80, 81] and have an active pentose-phosphate pathway [82, 83], these cells may be able to generate some ribose phosphates from pyruvate and lactate. In contrast, although the pure mitochondrial substrates acetate and β-hydroxybutyrate are efficiently consumed by astrocytes to fuel mitochondrial ATP regeneration [11–13, 84], these substrates will be fully oxidized by mitochondrial metabolism and can therefore not provide carbon for net synthesis of ribose phosphates.

The slow ATP restoration in BAM15-preincubated astrocytes after feeding of glucose was strongly accelerated by coapplication of adenosine leading to almost complete ATP restoration already within 60 min. The concentration dependency of the ATP restoration by adenosine revealed that maximal ATP restoration in the presence of glucose was already observed after 60 min incubation with 30 µM adenosine, demonstrating that the use of exogenous adenosine as substrate for the AMP synthesis that is needed for ATP restoration is highly efficient in the presence of glucose as energy substrate. For this condition a net synthesis of around 15 nmol/mg ATP was observed, accounting for the utilization of almost 30% of the extracellularly applied adenosine (7.5 nmol adenosine in 250 µL) as precursor for the AMP needed to restore ATP.

The adenosine-accelerated ATP synthesis was completely prevented by the presence of an inhibitor of adenosine kinase [85], demonstrating that adenosine can be efficiently phosphorylated in astrocytes to AMP which is needed as cellular precursor for further ATP restoration. In contrast, inhibitors of adenosine deaminase and/or purine nucleoside phosphorylase did not prevent the accelerated ATP restoration by adenosine, suggesting that synthesis of AMP via IMP and the purine nucleotide salvage pathway [60] are unlikely to contribute to the rapid generation of new AMP in ATP-deprived astrocytes after feeding of glucose plus adenosine.

Application of mitochondrial inhibitors revealed that mitochondrial oxidative phosphorylation is not required for the rapid ATP restoration in astrocytes in the presence of glucose plus adenosine, suggesting that the high rate of glycolytic metabolism of the glucose applied is sufficient to promote rapid ATP restoration in the presence of adenosine. This process does not require additional intracellular transport processes between compartments as the adenosine phosphorylating enzyme adenosine kinase is localized in the cytosol [58, 60].

Rapid but only partial ATP restoration was found for BAM15-pretreated astrocytes after application of adenosine as exclusive extracellular substrate. This cellular ATP restoration depended strongly on the adenosine concentration applied and the ATP level initially restored was only maintained in the presence of an additional energy substrate. This suggests for incubations with extracellular adenosine as exclusive substrate that the nucleoside serves both, as precursor for AMP synthesis and as energy substrate to fuel ADP phosphorylation. The presence of exogenous adenosine has previously been reported to prevent a loss in cellular ATP in starved astrocytes, but millimolar concentrations of adenosine were required to fully maintain a high ATP content for 24 h [13]. Of the adenosine molecule, only the catabolism of the ribose moiety has to be considered as fuel for energy production. Adenosine is phosphorolytically cleaved by purine nucleoside phosphorylase to adenine and ribose-1-phosphate [60, 69]. The ribose phosphate can then be further metabolized in astrocytes by the non-oxidative part of the pentose-phosphate pathway [82, 83] and by glycolysis to generate pyruvate, which can subsequently be transported into mitochondria and utilized as substrate to fuel mitochondrial oxidative phosphorylation. The contribution of adenosine catabolism in providing energy via ribose phosphates is supported by the potential of inhibitors of the adenosine metabolizing enzymes adenosine deaminase and purine nucleoside phosphorylase to lower ATP restoration from adenosine, if given as exclusive substrate. However, the rate of glycolytic ATP regeneration from adenosine-derived ribose phosphate appears to be too slow in astrocytes to fuel efficient ATP restoration, as inhibition of mitochondrial pyruvate uptake or of oxidative phosphorylation completely prevented ATP restoration from adenosine, while the formation and export of adenosine-derived lactate was found strongly accelerated for such conditions. The poor potential of extracellular adenosine to serve as substrate for glycolysis is also demonstrated by the low levels of basal lactate production and release in adenosine-treated astrocytes, which is at least one order of magnitude lower than the lactate release observed for glucose-treated astrocytes.

In summary, in order to study ATP restoration, cultured astrocytes were first depleted of most of their ATP by a preincubation without energy substrates in the presence of the uncoupler BAM15. Refeeding experiments revealed that this treatment did not compromise the ability of the cells to restore ATP. Astrocytes were found to be highly efficient to fully and rapidly restore their initial high cellular ATP content in the presence of glucose (or another energy substrate) plus adenosine as substrate for adenosine kinase-mediated phosphorylation to AMP.

ATP depletion in brain has been shown to rapidly occur in hypoxic and ischemic conditions [32, 86], in stroke [62, 87, 88] and traumatic head injuries [58, 89, 90]. Thus, it is of high importance to better understand the pathways that contribute to ATP restoration in such pathological conditions [32, 58, 61, 62]. As astrocytes have important functions in the brain as partners of neurons and as many astrocytic functions require ATP [7, 91–94], a better knowledge on ATP restoration in astrocytes may help to develop strategies to improve astrocytic ATP restoration in pathological conditions. This would help to reestablish important astrocytic functions which in turn would also help neighboring neurons and support the brain during recovery from insults that have lowered the ATP content in brain. However, a direct application of adenosine to foster ATP restoration in brain appears not to be appropriate as adenosine is highly neuroactive [58, 95–101]. Thus, further studies are now required to explore whether adenosine can be replaced by other nucleosides or purine bases for efficient ATP restoration in astrocytes and whether alternative combinations of energy substrates and AMP precursors may support an even better ATP restoration than the combination of glucose plus adenosine.

## Data Availability

Enquieries on original data should be directed to the corresponding author.
